# Melatonin serum level, sleep functions, and depression level after bilateral anodal transcranial direct current stimulation in patients with Parkinson’s disease: a feasibility study

**DOI:** 10.5935/1984-0063.20200083

**Published:** 2021

**Authors:** Hikmat Hadoush, Ansam Alqudah, Saleem A. Banihani, Muhammed Al-Jarrah, Akram Amro, Salameh Aldajah

**Affiliations:** 1 Jordan University of Science and Technology, Rehabilitation Sciences -Irbid - Irbid - Jordan.; 2 Jordan University of Science and Technology, Medical Laboratory Sciences - Irbid - Irbid - Jordan.; 3 Al-Quds University, Physiotherapy - Jerusalem - Jerusalem - Palestinian Territories.; 4 Isra University, Rehabilitation Sciences -Amman - Amman - Jordan.

**Keywords:** Parkinson’s Disease, Melatonin, Sleep, Depression, Transcranial Direct Current Stimulation

## Abstract

**Objective:**

Parkinson’s disease (PD) is associated with non-motor complications such as sleep disturbance and depression. Transcranial direct current stimulation (tDCS) showed therapeutic effects on the motor dysfunctions. However, the potential effects of tDCS therapy on melatonin hormone, sleep dysfunctions, and depression in patients with PD still unclear. This feasibility study aimed to identify any potential changes in melatonin serum level, sleep functions and depression after the bilateral anodal tDCS in patients with PD.

**Material and Methods:**

Tensessions of bilateral anodal tDCS stimulation applied over left and right prefrontal and motor areas were given to twenty-five patients with PD. Melatonin serum level, Pittsburgh sleep quality index, and geriatric depression scale examined before and after tDCS stimulation.

**Results:**

After bilateral anodal tDCS, there was a significant reduction in melatonin serum level, improvement in depression, improvements in overall sleep quality, and sleep latency. Correlations test showed significant associations between melatonin serum level reduction and changes in subjective sleep quality, and sleep duration, as well as between improvements in depression and overall sleep quality, sleep latency, and sleep disturbance.

**Conclusion:**

Bilateral anodal tDCS therapy was a feasible and safe tool that showed potential therapeutic effects on melatonin serum level, sleep quality, and depression level in patients with PD. Although the further large scale and randomized-control trial studies are crucially needed, there is still a need for such a feasibility study to be established before such trials can be implemented as is recommended in the new medical research council guidelines.

## INTRODUCTION

Parkinson’s disease (PD) is the second most common neurodegenerative disorder worldwide after Alzheimer’s disease^1^. A frequent non-motor symptom of PD such as depression and sleep dysfunction had a rate that reaches up to 90%^2,3,4^. The sleep dysfunctions and problems range from nocturnal or during the night issues such as difficulty with sleep initiation, sleep fragmentation and disruption, frequent nighttime awakening, disturbance of circadian rhythm, and rapid eye movement sleep behavior disorder, to the daytime problems such as excessive daytime sleepiness^5^. Diurnal fluctuations of motor and non-motor symptoms and high prevalence of sleep/ wake disturbances in Parkinson’s disease (PD) suggest a role of the circadian system in the modulation of these symptoms.

The sleep-wake cycle represents the most apparent circadian rhythm, and changes in this circadian amplitude and/or phase reported to reduce nighttime sleep quality, daytime alertness, and cognitive performance^6^. One of the multifactorial factors that regulate this circadian rhythm is the timing of melatonin secretion from the Pinal gland, and it is reported that PD participants with excessive daytime sleepiness had a significantly lower amplitude of the melatonin rhythm compared with PD participants without excessive sleepiness^7^.

On the other hand, PD is caused by the reduction in the neurotransmitter called dopamine^8^, which is an essential modulator of the basal ganglia. It is reported that apoptotic effects triggered by internal molecules or deleterious processes, such as oxidative stress, play a crucial role in the pathogenesis of PD^9,10^. Melatonin is an endogenous hormone that has recently been revealed as a potent antioxidant that could counteract oxidative stress. It has, thus, been hypothesized that it might be beneficial in the prevention or treatment of PD^11^. This is because studies have shown that melatonin also has a mutual regulatory effect with dopamine in the circadian cycle. During the day, melatonin level decreases and dopamine level increases, while at night, melatonin level increases and dopamine level decreases^12^. Therefore, regulation of circadian rhythm dysfunction may become a new target for therapeutic intervention. In the clinic, melatonin can be used as a potential biomarker reflecting the dysfunction of PD, providing new ideas for the early diagnosis and treatment of PD^13^.

Recently, transcranial direct current stimulation (tDCS) showed potential therapeutic effects in the management of non-motor symptoms in patients with PD such as working memory improvement^14^, sleep functions, depression perception level, and physical and mental quality of life^15^. Moreover, it was reported that tDCS influences brain-derived neurotrophic factor (BDNF) serum level in patients with PD, and this returned with a positive impact on the motor condition and stopped the dopamine serum level deterioration^16^. However, to our best knowledge, there are no studies investigated the effect of bilateral anodal tDCS on melatonin serum level and its potential correlation with the improvements in sleep and depression outcome measures in patients with PD.

Therefore, this study attempted to identify any potential therapeutic effect of bilateral anodal tDCS on the melatonin serum level, and its potential correlation to the changes in sleep functions and depression perception level in patients with PD.

## MATERIAL AND METHODS

### Study design

The study design and protocol were approved by the corresponding ethical committee and the Institutional Review Board (IRB) of the Jordan University of Science and Technology and it is registered in the clinical trial registry of the King Abdullah University Hospital (electronic No. 153/2019). Written informed consent was obtained from all patients before participation in this study, the confidentiality of information and anonymity of the participants was assured.

### Participants

During one year, a convenient sample of twenty-five patients with idiopathic PD (19 males and 6 females, aged 30 to 80-years-old) were recruited from public and private neurological clinics to participate in this study. They had stage I-V of modified Hoehn and Yahr scale during the “on stage” of medication, where stage I indicates unilateral involvement only, II indicates bilateral involvement without impairment of balance, III indicates mild to moderate bilateral disease, some postural instability, physically independent, and IV indicates severe disability, still able to walk or stand unassisted. The patients were not previously treated with any kind of brain stimulation (Table 1) and they had three weeks of stable medical regimen before the study and were capable of maintaining a stable regimen during the study period, the majority of participated patients were on SINEMET (carbidopa-levodopa) with dose ranges of 10-250mg.

**Table 1:** Demographic data.

N=25	Age (years)	H & Y stage
Females (n=6)	63.0 ± 9.5	3.0 ± 1.0
Males (n=19)	61.0 ± 9.3	2.0 ± 1.2

The table shows the demographics data of the participants’ gender, age, and disease stage according to Hoehn and Yare Scale (H & Y). Data presented as mean ± SD.

### Assessment & outcome measures

Three outcome measures were obtained before and after the tDCS stimulation including melatonin serum level, sleep functions, and depression perception level. First the melatonin serum level, venous blood samples were collected in plain tubes from participants before the tDCS therapeutic session and after the last tDCS session while they were in a sitting position. Blood collection time was standardized the blood collection time to be one-hour post the medication, and to be collected in the morning time from 8:30 to 10:30 a.m., and this time interval was in accordance with the previous study^17^.

Immediately after coagulation, the samples were centrifuged at 2000 × g, and serum was stored at -80 °C until analysis. Before the quantitation, serum samples were firstly prepared to detect melatonin levels using commercially available immunoassay kits (DRG Melatonin-Enzyme Linked Immunosorbent Assay, U.S.A). Briefly, each sample was passed through a reversed-phase column and the collected filtrate was extracted with methanol. Then, the extract was dried by evaporation and dissolved in deionized water. An immunoassay-based system was used for the quantitative determination of melatonin (Bio-TEK Instruments INC., Tokyo, Japan). All serum samples were assayed in duplicates and the mean values were considered for final statistical analysis.

Second, the sleep functions, the Pittsburgh sleep quality index (PSQI) was used to evaluate sleep quality measures before and after the bilateral anodal tDCS. The PSQI is a well-known, validated, and reliable self-reported instrument used to measure the clinical construct of sleep quality. It consists of 18 questions scored from 0 (no difficulty) to 3 (severe difficulty) and ends with the total score and sub-scores of 7 domains: 1) sleep quality, 2) sleep latency, 3) sleep duration, 4) sleep medication, 5) sleep disturbances, 6) daytime dysfunction, and 7) habitual sleep efficiency^18^.

Third, depression perception level, the geriatric depression scale (GDS) is a 30-item questionnaire, which is a highly reliable and valid screening tool used to measure the depression level across different ages, genders, and ethnicities in community and social service settings^19^.

### TDCS intervention

**Figure 1:**
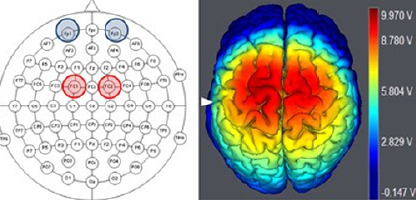
The tDCS electrodes placement. Brain schematic view shows the bilateral anodal tDCS electrodes locations, in which two anode electrodes placed over FC1 and FC2, and the two cathode electrodes placed over the left and right supraorbital area. Besides, a 3D brain image is structured by using our software (Neuroelecric, NIC, Spain) to show the simulated electric field distribution in the bilateral DLPFC, supplementary motor, and M1 areas that generated by the bilateral anodal tDCS stimulation. These electrodes placement is the same of previous work11.

The bilateral anodal tDCS stimulation was used based on the 10-10 EEG system to place two anodal electrodes over the left FC1 and right FC2, and the two cathodal electrodes were placed over the left and right Fp1 and Fp2 supraorbital area (Figure 1). This anodal electrodes’ placement over FC1 and FC2 was chosen to stimulate the primary motor area (M1) and the dorsolateral prefrontal area (DLPFC) of the right and left hemispheres simultaneously, and its efficacy has been confirmed in a previous study^20^, where the spatial distribution, magnitude, and direction of the current density was investigated in the computer-aided spherical head model during tDCS.

Both of the anodal and cathodal electrodes were 25cm^2^ surface area and moistened. The current intensity was 1mA.

Each patient received 10 sessions of bilateral anodal stimulation for 20min per session (5 sessions per week for 2 weeks). This stimulation dosage and protocol were reported to have no adverse or side effects in patients with PD^15,21^.

### Statistical analysis

A paired t-test was used to identify any potential significant difference between the outcome measures before and after the bilateral anodal tDCS. Besides, Pearson’s correlation test was used to identify any potential correlation between the outcome measures changes.

## RESULTS

**Figure 2:**
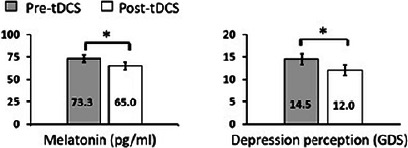
Melatonin serum level and depression perception level changes. The left graph shows a comparison between the measured melatonin serum level pre-tDCS treatment (73.2±4.4pg/ml) and post-tDCS treatment (65±4.0pg/ml), where * indicates significant difference at p=0.037. The right graph shows a comparison between the depression perception level (GDS score) pre-tDCS treatment (14.56±1.20) and post-tDCS treatment (12.0±1.18), where * indicates significant difference at p=0.028.

There was a significant decrease in the mean value of the melatonin serum level after bilateral anodal tDCS therapy (*p*=0.043) with small to moderate effect size (Cohen’s d=0.40), as well as there was a significant decrease in the mean value of depression levels (GDS) after bilateral anodal tDCS therapy (*p*=0.027) with a small to moderate effect size (Cohen’s d=0.42) (Figure 2).

**Figure 3:**
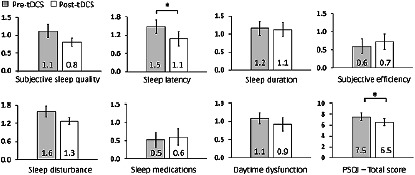
Sleep function changes. Graph shows the comparison between the calculated PSQI total score and sub-scores before and after the bilateral anodal tDCS stimulation. *Indicates significant difference with p-values as the following; PSQI - Total score mean value pre-tDCS (7.5±0.66) and post-tDCS (6.5±0.61) with p=0.028, and sleep latency mean value pre-tDCS (1.5±0.22) and post-tDCS (1.1±0.23) with p=0.021. All other sleep functions parameters shows no statically significant difference (p>0.05).

In terms of sleep functions, there was a significant improvement in the overall sleep quality (PSQI total score, *p*=0.028) with a small effect size (Cohen’s d=0.31), as well as there was a significant improvement in the sleep latency (PSQI sub-score, *p*=0.022) with a small effect size of (Cohen’s d=0.35). Although the reset PSQI sub-scores including sleep duration, sleep medication, sleep disturbances, daytime dysfunction, and habitual sleep efficiency showed different improvements, the statistical significances were not obtained (Figure 3).

**Figure 4:**
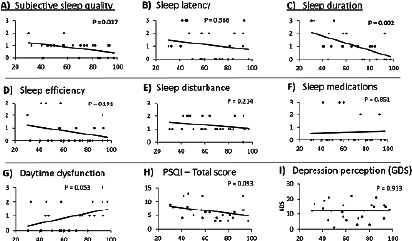
Correlation between melatonin serum level, sleep functions, and depression level. Scatter plotting figures show the correlation between melatonin serum level (X-axis), and sleep function outcome measures including the PSQI total score and sub-scores, and the depression perception level (GDS score) after the bilateral anodal tDCS treatment. Pearson's correlations test showed significant correlations between melatonin serum level reduction, and the improvement in subjective sleep quality subscore (A) (Rho=-0.419, p=0.037), and the sleep duration sub-score (C) (Rho=-0.585, p=0.001). However, melatonin serum levels had weak and insignificant correlations with sleep latency, sleep efficiency, and sleep disturbance, sleep medication, daytime dysfunction, PSQI total score, and depression perception level (GDS score).

On the other hand, Pearson’s correlations test showed significant correlations between melatonin serum level reduction, and the improvement in subjective sleep quality (Rho=-0.419, *p*=0.037), sleep duration (Rho=-0.585, *p*=0.001). However, melatonin serum levels had weak and insignificant correlations with sleep latency, sleep efficiency, and sleep disturbance, use of sleep medication, daytime dysfunction, PSQI total score, and depression level (Figure 4).

**Figure 5:**
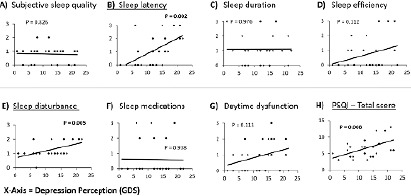
Correlation between sleep functions and depression level. Scatter plotting figures show the correlation between the depression perception level (GDS score, X-axis) and the sleep functions outcome measures including the PSQI total score and sub-scores after the bilateral anodal tDCS treatment. Pearson's correlations test showed significant correlations between the improvement in depression perception level (GDS score) and the sleep latency sub-score (B) (Rho=0.597, p=0.002), the sleep disturbance sub-score (E) (Rho=0.541, p=0.005), and the overall sleep quality (H) (PSQI total score, Rho=0.516, p=0.008). However, the improvement in the depression perception levels (GDS score) had weak and insignificant correlations with subjective sleep quality, sleep duration, sleep efficiency, sleep medication, and daytime dysfunction sub-scores.

In addition, Pearson’s correlations test showed significant correlations between the depression level and overall sleep quality (PSQI total score, Rho=0.516, *p*=0.008), sleep latency (Rho=0.597, *p*=0.002), and sleep disturbance (Rho=0.541, *p*=0.005). However, depression levels had weak and insignificant correlations with subjective sleep quality, sleep duration, sleep efficiency, use of sleep medication, and daytime dysfunction (Figure 5).

## DISCUSSION

In terms of melatonin serum level and sleep function changes, the melatonin serum level in patients with PD compared with the healthy individuals, and its correlation with sleep dysfunctions still controversial and unclear. For example, Willis (2008)^22^, reported that there is abundant are ample evidences describing the PD as an endocrine disorder of melatonin overproduction, whereas Videnovic et al. (2014)^7^ reported that patients with PD who suffer from excessive daytime sleepiness had a significantly lower amplitude of the melatonin serum level. However, in the same year 2014, Lin et al. (2014)^17^ reported that the melatonin serum levels in the morning either in the animal PD model or patients with PD were significantly higher than that the melatonin serum level of the control group. Besides, they demonstrated that melatonin serum levels are positively correlated with the severity of the PD according to measure by the Hoehn and Yahr scale. Later on in 2020, Li et al. (2020)^13^ reported that the plasma melatonin levels in PD patients were significantly higher than those in healthy controls, besides they reported that both sleep dysfunctions and disorders had a significant negative correlation with plasma melatonin levels.

The discrepancies findings and controversy also extended to the clinical studies examined melatonin therapy and the few studies that examined the electrical stimulation effects on the melatonin level. For example, Dowling et al. (2005)^23^ and Medeiros et al. (2007)^24^ conducted a trial on patients with PD and sleep dysfunctions and problems, where they divided them into two groups of the melatonin treatment group vs. placebo group. Then, they reported improvements in the different sleep functions and domains including subjective sleep disturbance, sleep quality, and daytime sleepiness, however, the reported improvement was dose-related. Besides, Belaid et al. (2015)^25^ reported an improvement in sleep parameters of melatonin treatment in PD monkeys. However, Gilat et al. (2020)^26^, examined the effect of melatonin treatment over the REM behavioral disorders in patients with PD and reported that there were no significant changes between the treatment group and the placebo group.

In addition, in animal PD models, therapeutic interventions and approaches that increase the bioavailability of melatonin in the bloodstream appear to increase the severity of Parkinson’s symptoms, whereas therapies that reducing the melatonin serum level by pinealectomy or exposure to bright light can improving the parkinsonism symptoms^27,28^. Consequently, several studies^29,30,31^ introduced the light-based therapy that assumed to reduce the melatonin serum level, and they reported that patients with PD exposed to fluorescent light therapy at an intensity ranging between 1,000-10,000 lux for 1-1.5 hours for a various period showed improvement in mood status, sleep functions, insomnia, and depression perception level.

On the other hand, Zisapel e Laudon (1982)^32^ were the first to dissect out the animal hypothalamus and exposing it to two successive sets of electrical field stimulation, and they reported a reduction in melatonin secretion.

Whereas, Reuss et al. (1985)^33^ conducted a study on animal models where they exposed the hypothalamic paraventricular nuclei to an electrical current, and they concluded that melatonin tends to be insignificantly reduced. However, in human studies, Catala et al. (1997)^34^ administered a deep brain stimulation to the internal globus pallidus of patients with PD, and they found that patients with PD showed decreased melatonin level after deep brain stimulation, but the mechanism behind the decrease in melatonin after their electrical stimulation is not reported. Therefore, based on the electrophysiological, tDCS, and targeted cortical regions we could assume two scenarios and mechanisms stand behind our findings.

First, in the case of patients with PD had higher melatonin serum levels as reported in previous studies^16,21,22^, and reduction of the melatonin would return with potential therapeutic effect^29,30,31^, then the following scenario would be possible. The anodal tDCS over the DLPFC reported to activate and increase striatal glutamate levels when measured with magnetic resonance spectroscopy in healthy adults^35^. In addition, Villela et al. (2013)^36^ reported that melatonin synthesis is suppressed by the activation of the glutamate receptors through decreasing the content of N-acetylserotonin (the immediate precursor for melatonin synthesis), which in turn, decreases the melatonin synthesis and its level in the central nervous system, as well as its mRNA expression. Therefore, we assumed that bilateral anodal tDCS therapy applied over left and right DLPFC, premotor, and primary motor areas would decrease the melatonin serum level by enhancing the striatal glutamate activity in patients with PD, which in turn would improve the sleep functions and depression levels. However, this study design cannot either confirm or deny this mechanism and further studies are needed.

Second, the improvements in sleep functions and depression in this study would be a direct result of the applied bilateral anodal tDCS stimulation over the DLPFC, premotor, and primary motor areas. This is because in terms of sleep functions both left and right DLPFC, supplementary motor area, premotor, and M1 are reported to be affected in PD^37^. Besides, an EEG^38^ and brain topography^39^ studies reported that both the right and left prefrontal and, DLPFC, premotor, and primary motor areas are all responsible for and involved in the sleep functions^38^. Therefore, this study’s bilateral anodal tDCS stimulation protocol over both left and right DLPFC, premotor, and M1 areas is assumed to serve as a comprehensive stimulation protocol that assures the activation of the major cortical areas involved in sleep regulation. In turn, bilateral anodal tDCS could improve the sleep quality measures (PSQI total score, and sleep latency) in patients with PD.

Whereas, in terms of depression, it was reported that depression comes with a significant diminishing in the DLPFC activity levels^40^, in both supplementary motor area and M1 area^18^. Consequently, previous tDCS studies concluded that tDCS could show antidepressant effects in patients with neurological diseases including PD^41,42^. In addition, neuroimaging studies reported that there is an association between sleep disturbance and sleep latency, and depression in various neurological disorders including PD^43,44,45^. Therefore, we assumed the significant decrease in depression level after bilateral anodal stimulation, over DLPFC, premotor, and M1 areas, as a result of the direct effect of tDCS stimulation, or/and a result of the indirect effect of sleep quality improvement. In turn, bilateral anodal tDCS could improve the depression measure GDS in patients with PD.

On the other hand, our data showed that decreased melatonin serum level is associated with improved sleep duration, which in turn, is associated with improved depression perception level after bilateral anodal tDCS in patients with PD. These associations are in line with several studies’ findings^44,46^. Melatonin was found to be correlated with sleep duration in patients with hypertension who suffer from nighttime sleep disturbances^46^. Besides, reduced sleep duration was found to be correlated with poor sleep quality in pregnant women^47^; and, sleep duration is correlated with depression levels in patients with PD^44^. Therefore, this study’s bilateral anodal tDCS over the cortical areas involved in depression and sleep regulation, and/or the improvements in sleep quality and depression level measures could influence the reduction in melatonin serum level in our patients with PD. However, future studies are needed to explain the mechanism behind the involvement of melatonin in the improvement of sleep and depression in patients with PD.

## CONCLUSION

Bilateral anodal tDCS was a safe stimulation protocol that showed therapeutic effects on melatonin serum level, sleep functions, and depression perception level in patients with PD as well as associations between these changes was found. However, several limitations should be considered regarding the study findings including the relatively small sample size, lacking the data from the healthy control group, examining the melatonin serum level at one point instead of 24-hours, and recruiting patients with approximately similar age group.

Therefore, future study with larger sample size, and matched healthy control group, and with longitudinal measurement is needed. Besides, including outcome measures regarding the motor function and dopamine serum level in parallel to the melatonin serum level, and testing and recruiting patients with different age groups would be essential to understand such endocrine and neurotransmitters interaction networks. However, there is still a need for such a feasibility study to be established before such trials can be implemented as is recommended in the new medical research council guidelines^48^.
